# The apolipoprotein gene: a modulating role on brain volume and cognitive function in carriers of the fragile X premutation

**DOI:** 10.1016/j.nbd.2026.107292

**Published:** 2026-02-02

**Authors:** Poonnada Jiraanont, Jun Yi Wang, Blythe Durbin-Johnson, Ye Hyun Hwang, David Hessl, Susan M. Rivera, Randi J. Hagerman, Flora Tassone

**Affiliations:** a Division of Molecular and Cellular Medicine, Faculty of Medicine, King Mongkuťs Institute of Technology Ladkrabang, Bangkok, Thailand; b Department of Biochemistry and Molecular Medicine, School of Medicine, University of California Davis, Sacramento, CA, USA; c Department of Pediatrics, University of California Davis, School of Medicine, Sacramento, CA, USA; d MIND Institute, University of California Davis Health, Sacramento, CA, USA; e Center for Mind and Brain, University of California Davis, Davis, CA, USA; f Division of Biostatistics, University of California Davis, School of Medicine, Davis, CA, USA; g Department of Psychiatry and Behavioral Sciences, University of California Davis, School of Medicine, Sacramento, CA, USA; h Department of Psychology, University of Maryland, College Park, MD, USA

**Keywords:** FXTAS, Premutation, *Apoε*, Motor function, Brain volume

## Abstract

Fragile X-associated tremor/ataxia syndrome (FXTAS), caused by the *FMR1* premutation allele, is associated with brain degeneration, yet the mechanisms behind this neurodegeneration still need to be elucidated. *Apoε* polymorphism has been widely implicated in brain aging in cognitively healthy individuals and brain deterioration in Alzheimer's disease. This study aimed to examine the interaction of *Apoε* genotypes, FXTAS clinical symptoms, *FMR1* molecular measures, and age, towards brain pathophysiology and cognitive functions.

This longitudinal study includes MRI data collected from 205 male premutation carriers with and without FXTAS clinical symptoms and compared to 86 healthy male controls aged 40–85 years. The investigation includes FXTAS-related brain volumes, IQ, self-control behaviors, *FMR1* molecular measures, and *Apoε* genotypes.

In carriers with FXTAS, the presence of the *Apoε2* allele showed a possible association with more favorable neuroimaging markers, such as reduced white matter hyperintensities, and lower incidence of the middle cerebellar peduncle sign, patterns that were not observed in carriers without FXTAS.

Specifically, the presence of *Apoε2* allele exhibited a potential protective effect on brain degeneration, and cognitive functions among FXTAS patients; on the contrary, the *Apoε4* allele was associated with a worsening of brain volume and brain degeneration in carriers with no FXTAS symptoms. The identification of *Apoε* genotypes in *FMR1* premutation carriers before any clinical symptoms of FXTAS are observed may improve symptomatic management leading to better outcomes for these individuals.

## 1. Introduction

Fragile X-associated tremor/ataxia syndrome (FXTAS) is a late-onset progressive neurodegenerative disorder manifested among carriers with 55–200 CGG repeat expansion in the 5′ untranslated region of the fragile X messenger ribonucleoprotein 1 (*FMR1*) gene (Hagerman et al., 2001; Jacquemont et al., 2003; Juncos et al., 2011; Apartis et al., 2012). FXTAS has been reported to occur in approximately 40–75% of male and 8–20% of female carriers of the *FMR1* premutation, with the risk increasing with age (Jacquemont et al., 2004; Cabal-Herrera et al., 2020; Leehey, 2009). They present with clinical core features including intention tremor, gait ataxia, executive dysfunction, and memory deficits (Hagerman, 2013; Hagerman and Hagerman, 2015). It is believed that increased *FMR1* mRNA demonstrated in those with FXTAS leads to RNA toxicity and ultimately to neurodegeneration (Tassone et al., 2001). Male premutation carriers (PC) with FXTAS, particularly those with mid-ranged CGG repeat alleles (80–100 repeats), show reduced cerebellar, brainstem and whole brain volumes and larger ventricular volumes suggesting the mid-ranged CGG repeats might be a considerable predictor of volumetric decline in PC (Hessl et al., 2025; Wang et al., 2017). A longitudinal study of ventricle enlargement revealed that male PC with and without FXTAS had enlarged fourth ventricles and those with FXTAS exhibited significantly enlarged third and lateral ventricles. The authors proposed that, with age, ventricular enlargement progressed from the fourth ventricle to the third ventricle and finally to the lateral ventricles in FXTAS (Wang et al., 2020). Further, enlarged perivascular spaces (PVS), indicators of glymphatic abnormalities, have been reported to play a role in cognitive deterioration (Staals et al., 2015). Interestingly, male PC with FXTAS exhibited substantially enlarged PVS in the basal ganglia, which correlated with enlarged ventricles, increased white matter lesions, brain atrophy, and decreased global cognitive function. In addition, PC with FXTAS and with the middle cerebellar peduncle (MCP) sign, a common and characteristic sign for FXTAS (Hagerman and Hagerman, 2013), exhibited significantly higher CGG repeat numbers than FXTAS carriers without the MCP sign (Elias-Mas et al., 2024). These findings indicated that the cerebellar-basal ganglia circuitry is damaged in FXTAS, and the damage likely results from CGG expansion-induced RNA toxicity. Both the basal ganglia and the cerebellum are important for movement and cognitive functions. They reciprocally interconnect with the cerebral cortex, forming multisynaptic loops involved in classic sensorimotor functions as well as reward-enhancing associative learning of movements (Wagner and Luo, 2020; Yoshida et al., 2022) functions especially affected in carriers with FXTAS (O'Keefe et al., 2015; O'Keefe et al., 2018; Brown et al., 2018).

The Apolipoprotein E (*Apoε*) is a long well-documented lipoprotein playing a key role in neurodegenerative disorders particularly Alzheimer's disease (AD) (Farrer et al., 1997; Neu et al., 2017; Yamazaki et al., 2019; Raulin et al., 2022; Jackson et al., 2024) and has been implicated in white-matter integrity across aging and neurodegenerative conditions (Belaidi 2025). *Apoε* is a principal transporter of lipoproteins and a key component of lipoprotein complexes playing a multifunctional role in the homeostasis of cholesterol and of other lipid soluble molecules in the central nervous system (Mahley et al., 2009; Ferreira et al., 2011). Neurons and supporting cells utilize *Apoε,* abundantly involved in physiological, pathological, and cellular damage conditions (Boyles et al., 1985; Pitas et al., 1987; DeMattos et al., 2001). The *Apoε* gene produces 299 amino acids and is located on chromosome 9q13.3 with three primary human isoforms: *Apoε2, Apoε3*, and *Apoε*4 (Weisgraber and Shinto, 1991). The *Apoε4* represents the strongest genetic risk factor for AD, while the *Apoε2* has shown to be protective in AD against amyloid (Aβ) accumulation in the regions of the brain associated with cognitive impairment. The neutral *Apoε3* is the most common allele in all ethnic groups (Bertram and Tanzi, 2008; Vemuri et al., 2010; Holtzman et al., 2012; Liu et al., 2013; Reiman et al., 2020). The *Apoε* isoforms differ meaningfully in their lipidation status and receptor interactions, such that *Apoε4* is less efficiently lipidated and thus poorer at transporting cholesterol and phospholipids essential for myelin membrane maintenance compared to *Apoε3* and *Apoε2* isoforms. This impaired lipid transport contributes to vulnerability of myelin and oligodendrocyte populations, and has been linked to exacerbated myelin breakdown and reduced numbers of actively myelinating oligodendrocytes as shown in animal models and human imaging studies of aging and Alzheimer's disease (AD) risk alleles (Yang et al., 2023).

Human imaging and histopathological studies have related *Apoε* genotype to differential white-matter microstructural trajectories, reflecting the health of the brain's communication highways, bundles of myelinated axons that connect different brain regions and support cognitive and motor function. Carriers of the protective *Apoε2* allele exhibit a relative preservation of myelin integrity and slower age-related myelin breakdown compared with *Apoε4* carriers, as measured by diffusion and relaxation MRI metrics in cognitively normal older adults, supporting genotype-dependent effects on myelin stability in late-myelinating association tracts (Bartzokis et al., 2006).

In order to identify a potential sensitive biomarker for susceptibility of AD, numerous studies (Lyall et al., 2020; Chaloemtoem et al., 2024; Heise et al., 2024; Veldsman et al., 2021; Lim et al., 2017) examined the role of *Apoε4* together with age and sex in brain volumes, and cognitive abilities among healthy normal population (the UK Biobank). A cross-sectional population-based study (Lyall et al., 2020) investigated how *Apoε4* is associated with brain imaging measurements and age in 8395 participants. Results demonstrated that *Apoε4* was significantly associated with greater white matter hyperintensity (WMH) volumes but not with hippocampal volumes, total gray matter or white matter volumes, and age. The authors suggested that *Apoε4* is related to poor cerebrovascular health during cognitive aging. Recently, the interaction between *Apoε* genotypes, age, and hippocampal volume (Chaloemtoem et al., 2024) was further examined in 37,463 individuals age ranged from 44 to 82 years. Hippocampal volume reduction was detected the earliest (at age 61) in those with the *Apoε4/ε4* genotype and eight years later (at age 69) in those with the *Apoε3/ε4*, while those with the *Apoε2/ε3* allele genotype had deferred hippocampal volume loss with decrements beginning at 76 years of age. This work emphasized the significant interaction between *Apoε* genotypes and age on hippocampal volume trajectories in the general population. The importance of the interplay between *Apoε* genotypes with age and sex (Heise et al., 2024) was also investigated on gray and white matter structures in 28,494 cognitively healthy individuals. The findings also demonstrated that volume of WMH was robustly greater in individuals with the *Apoε4/ε4* and *Apoε3/ε4* genotypes, but not in those with *Apoε2* and *Apoε3/ε3*. In addition, the white matter integrity of four tracts, including the dorsal and ventral cingulum, posterior thalamic radiation, and sagittal stratum, exhibited less integrity among those with *Apoε4/ε4* and *Apoε3/ε4* genotypes compared with those with *Apoε2* and *Apoε3/ε3* genotypes. Interestingly, after age 60 years, those with the *Apoε4/ε4* genotype demonstrated decreased white matter integrity that was comparable with other genotypes at 5 years greater age, indicating accelerated aging process in older *Apoε4/ε4* carriers. This work suggested that the *Apoε4* genotype influences white matter integrity particularly after 60 years of age. Taken all together, these studies strongly suggested that the *Apoε4* genotype is associated with brain pathophysiology in individuals who are 60 years or older.

Intriguingly, one study revealed a high prevalence of at least one *Apoε4* allele among 44 male carriers with FXTAS (8/22; 36.3%) compared to carriers without FXTAS (1/22; 4.5%) suggesting that the *Apoε4* allele may confer increased risk for early FXTAS onset (Silva et al., 2013). However, a more recent study (Winarni et al., 2024) reported a 22% prevalence of the *Apoε4* genotypes among 245 male carriers with FXTAS and found no association with FXTAS stage, suggesting that *Apoε4* may not contribute to FXTAS development or severity.

Importantly, although direct *Apoε* associations with FXTAS phenotypes (e.g., CGG repeat length) are not reported, the pathological hallmark of FXTAS is profound white-matter degeneration, including spongiosis and loss of axons and myelin particularly in the MCP and subcortical tracts visualized as hyperintensities on MRI confirmed at autopsy (Tassone et al., 2023). Given the extensive evidence that *Apoε* allelic variation can modulate white-matter microstructure, lipid homeostasis, oligodendrocyte support, and glial responses, processes central to myelin maintenance and repair, there is strong biological plausibility that *Apoε*-mediated pathways could influence the severity or progression of white-matter degeneration in FXTAS carriers independent of the CGG repeat effects. These pathways may operate through differential lipid transport to oligodendrocytes, altered cholesterol handling during remyelination, and isoform-specific glial inflammatory responses, all of which have been demonstrated in both human and model systems as modifiers of white-matter vulnerability in aging and neurodegenerative contexts (Yang et al., 2023).

Here, we report on a study investigating whether the *Apoε* genotype and *FMR1* molecular measures (i.e. CGG repeat size, AGG interruptions, repeat instability, and *FMR1* mRNA levels) along with age and FXTAS status, contribute to neurodegeneration and cognitive impairment in FXTAS.

## 2. Material and methods

### 2.1. Participants

Male research participants were recruited between 2007 and 2023 by 2 ongoing longitudinal studies. The studies were conducted following procedures approved by the University of California Davis Institutional Review Board with IRB number 254134–33 and 473010. All participants provided written informed consent before participation in line with the Declaration of Helsinki. Inclusion criteria were the confirmed carriers of fragile X premutation by genetic testing (*FMR1* CGG repeat length between 55 and 200). All participants were aged 40 years or older without any life-threatening illness other than FXTAS. *Apoε* genotypes were determined from 86 male controls with normal *FMR1* alleles and 205 male PC. FXTAS was diagnosed by trained physicians based on core symptoms of FXTAS, including the presence of intention tremor, cerebellar ataxia, cognitive impairment, and white matter lesions in specific brain regions (Jacquemont et al., 2004; Hall et al., 2014). PC were categorized by FXTAS diagnosis, with FXTAS diagnosis of “No” defining the “FXP−” group and FXTAS diagnosis of “Possible”, “Probable”, and “Definite” defining the “FXP+” group.

Cognitive functioning was assessed using Wechsler Adult Intelligence Scale, Third (WAIS III, 173 participants, 271 visits), and Fourth editions (WAIS IV, 79 participants, 111 visits) and Wechsler Abbreviated Scale of Intelligence, Second Edition (WASI-2, 71 participants, 100 visits) (Wechsler, 1997; Wechsler, 2008; Wechsler, 2011). Full scale IQ (FSIQ) from all editions and Working Memory Index (WMI) and Processing Speed Index (PSI) from WAIS III and IV were included in the analyses. Motor and attentional self-regulation was evaluated using the Behavioral Dyscontrol Scale-Second Edition (BDS-2) (Grigsby et al., 1992; Shura et al., 2015). Dexterity was tested using the total score of Purdue Pegboard (Tiffin and Asher, 1948).

### 2.2. Molecular measures

Genomic DNA was isolated from peripheral blood lymphocytes using a standard method (Qiagen, Valencia, CA, USA). CGG trinucleotide repeat size and methylation status were determined using a combination of PCR and Southern blot analysis as previously described (Tassone et al., 2008; Filipovic-Sadic et al., 2010). The percentage of methylation was measured by densitometry analysis (Tassone et al., 1999). Male carriers with either one or two premutation alleles, and no evidence of methylation, were included in the study. For PC with two *FMR1* premutation alleles, CGG repeat size was averaged.

Total cellular RNA was purified from 2.5 ml peripheral blood. *FMR1* mRNA expression levels were measured by real time qRT-PCR as previously described (Tassone et al., 2000). The characterization of the *Apoε* genotypes was carried out by single nucleotide polymorphism (SNP) analysis utilizing 50 ng of gDNA, and two TaqMan probes, rs429358 and rs7412, were used for the assay (Applied Biosystems, Inc., Foster City, CA, USA). CGG repeat instability, defined as somatic variation in the length of the *FMR1* CGG tract, with the presence of a series of expanded alleles that differ from the next allele by a single repeat unit, was quantified following the method of Hwang et al. (Hwang et al., 2022) PCR amplicons were separated by capillary electrophoresis, and the resulting CE traces were analyzed with Gene Mapper software to characterize the distribution of repeat sizes.

### 2.3. MRI acquisitions

Standard high-resolution T1-weighted magnetization-prepared rapid gradient-echo images and standard fluid-attenuated inversion recovery (FLAIR) images were acquired on a Siemens Trio 3 T MRI scanner with a 32-channel head coil (Siemens Medical Solutions, Erlangen, Germany). Of the 510 scans included in the study, 5 (1%) scans were acquired using the Siemens Trio system with an 8-channel head coil, 484 (95%) scans were acquired using the Siemens Trio system with a 32-channel head coil, and 21 (4%) scans were obtained using the Siemens Prisma system with a 32-channel head coil. Only scans that contained minimum motion artifacts not interfering with further processing were selected for volume quantification. Two premutation carriers who had only one visit were excluded because of excessive motion in their T1 scans, and the FLAIR scan obtained during the first visit of another premutation carrier was also excluded for the same reason. The acquisition protocols for T1/FLAIR were repetition time of 2180/5000 ms, echo time of 4.86/457 ms, inversion time of 1100/1700 ms, flip angle of 7°/120°, field of view of 256 × 256/256 × 256 mm^2^, voxel size of 1 × 1 × 1/0.95 × 0.95 × 1.9 mm^3^, and averages of 1/1.

### 2.4. MRI analyses

Volumes of the whole brain, cerebellum, brainstem, and lateral ventricles and brain scaling factor (Buckner et al., 2004) for adjusting individual differences in cranial size were generated using T1 images via the automated pipeline, vol2Brain (Manjón et al., 2022). Vol2Brain used multi-atlas label fusion technique to generate labels and patch-based neural networks for correcting systematic errors in the labels. The preprocessing steps included denoising, correction for inhomogeneity, affine registration to the Montreal Neurological Institute space and image intensity normalization (Manjón et al., 2022). After intracranial cavity extraction, the whole brain was segmented using a non-local patch-based label fusion technique. Labels were determined by searching for patches with similar patterns in the training library and random errors in the labels were minimized by averaging the votes of many patches. Systematic errors in the labels were corrected using a patch-based ensemble of neural networks trained with two types of image patches: (1) fully sampled patches of 3 × 3 × 3 voxels and (2) 5/7 sampled patches of 7 × 7 × 7 voxels. Ten neural networks were trained using a boosting strategy that prioritized high probability in misclassification when selecting training data for a new network.

**Fig. 1.**
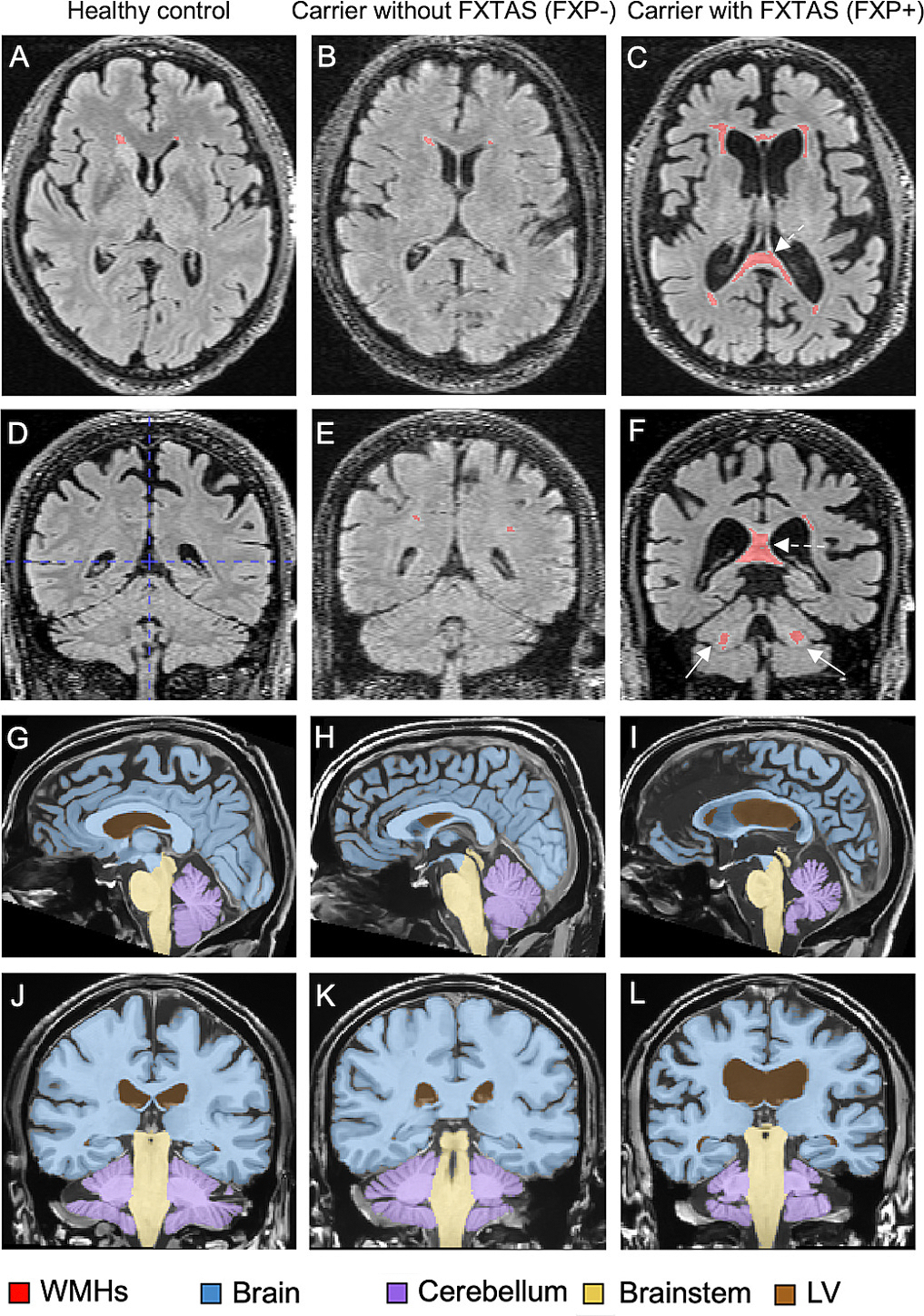
Representative segmentations of white matter hyperintensities (A–F), whole brain (G–L), cerebellum (G–L), brainstem (G–L), and lateral ventricles (LV, G–L) in a healthy control (A, D, G, & J), a premutation carrier without FXTAS (B, E, H, & K), and a premutation carrier with FXTAS (C, F, I, & L) all at the age of 62 years. (A–C) Axial views of the FLAIR scans showing the segmented white matter hyperintensities including the splenium sign of the corpus callosum (dash arrow) in C. (D–F) Coronal views of the FLAIR scans showing the segmented white matter hyperintensities including the splenium sign of the corpus callosum (dash arrow) and the MCP sign (straight arrows) in F. (G–I) Sagittal views of the T1 images showing the segmentation of the brain, cerebellum, brainstem, and LV. (J–L) Coronal views of the T1 images showing the segmentation of the brain, cerebellum, brainstem, and LV.

The generated 204 labels were combined using FSL (Smith et al., 2004) to form 18 labels covering the whole brain. We then used a machine-learning-based tool, SegAdapter (Wang et al., 2011), to correct additional systematic errors, and used ITK-Snap (Yushkevich et al., 2016) to manually correct residual errors blind to the status of the participants (Hessl et al., 2025). Whole brain WMH volume was quantified using both FLAIR and T1 images following the previously published methods (Wang et al., 2021). WMHs appear as bright spots with clear boundaries on MRI and indicate areas where white matter may be damaged or demyelinated, impacting brain connectivity. Briefly, the masks of WMHs were generated using the open-source tool, Lesion Segmentation Toolbox (LST) (Schmidt et al., 2019). Errors in the WMH masks were corrected by thresholding using the fslmaths command from FSL followed by manual correction using ITK-Snap blind to the status of the participants. Volume calculation was conducted using the command tools, fslmaths and fslstats, from FSL. Fig. 1 shows the representative segmentation of the WMHs and the whole brain, cerebellum, brainstem, and lateral ventricles in a healthy control, an FXP−, and an FXP+, all at age 62 years. Presence of the MCP sign was determined visually based on the WMH segmentation generated automatically by the LST tool. The MCP sign reflects a key region of white-matter degeneration typical in FXTAS and helps identify disease-related neurodegeneration visually on MRI.

### 2.5. Statistical analysis

#### 2.5.1. Brain measures with Apoε genotypes

Statistical analyses were conducted using the open-source R software, version 4.4.1 (R: A Language and Environment for Statistical Computing, 2023). For each type of statistical analyses, the Benjamini-Hochberg's false discovery rate (FDR) procedure was employed to control for multiple testing (Benjamini and Hochberg, 1995). Missing data were excluded from the analyses.

For cross-sectional group comparisons of participant characteristics at last visit, linear regression was used for comparing differences in age between the groups of controls, FXP−, and FXP+. Linear regression was used for comparing group differences in FSIQ, WMI, and PSI, with age, IQ test version, and years of education as covariates. For non-normal data including years of education, BDS-2, and dexterity scores, the Kruskal-Wallis test was performed to study the group differences followed by post-hoc pairwise comparisons using Wilcoxon rank-sum tests. Group differences in proportions of hypertension and cognitive impairment at last visit were tested using Fisher's exact tests.

Cross-sectional group differences in the frequencies of *Apoε2* and *Apoε4* genotypes at last visit were compared using binomial logistic regression adjusted for age. The effect of the *Apoε* genotypes (i.e., *Apoε2* and *Apoε4*) and their interactions with age on individual longitudinal MRI measures of neurodegeneration were assessed using mixed-effects models with hypertension as a covariate and a random intercept for participant. The analyses were conducted separately in each of the three groups. The interaction term was included only if significant. Volumes of WMHs and lateral ventricles were log transformed prior to statistical analyses to meet the normality assumption. The effects of the *Apoε2* and *Apoε4* genotypes and their interactions with age on the presence of the MCP sign were examined similarly using generalized linear mixed-effects models in the FXP+ group with hypertension as a covariate and a random intercept for participant. The effect of *Apoε* genotypes and their interactions with age on individual cognitive scores were also assessed similarly using mixed-effects models with a random intercept for participant including FXP+. BDS-2 scores were squared prior to statistical analyses to meet the normality assumption. Finally, we also explored independent effects of *Apoε* genotypes and CGG repeat length on cognitive functioning in the FXP+ using mixed-effects models adjusted for age with a random intercept for participant. A brain scaling factor was included as a covariate for all mixed-effects models involving the MRI data and years of education and IQ test version were included as a covariates for all mixed-effects models involving FSIQ, WMI, and PSI.

#### 2.5.2. The effect of FMR1 molecular measurements on brain MRI data

Continuous brain measures in *FMR1* PC were modelled by FXTAS stage using multivariable linear mixed effects models that included fixed effects for FXTAS stage, CGG repeats, age at brain scan, and brain scaling factor, and a random intercept for participant. Continuous brain measures were modelled by somatic expansion using multivariable linear mixed effects models that included fixed effects for instability, CGG repeats, age at brain scan, and brain scaling factor, and a random intercept for participant. Continuous brain measures were log transformed prior to analysis. The MCP sign was modelled by FXTAS stage, by CGG repeat size instability, and by *Apoε* genotypes using binomial generalized estimating equations (GEE) (Liang and Zeger, 1986). GEE models used the same fixed effects as models for continuous brain measures described above, with participant as cluster. GEE fits used an independence working correlation structure, with standard errors based on a sandwich estimator that is robust to misspecification of the working correlation (Liang and Zeger, 1986). FXTAS stage (grouped as 0/1, 2, 3, and 4/5) was modelled by subject characteristics using a multivariable mixed effects proportional odds logistic regression model (Agresti, 2010), with fixed effects for CGG repeats, *FMR1* mRNA, AGG, instability, and age at brain scan, and a random effect for participant. Analyses used the lowest CGG repeat reported for participants with a range or smear. Somatic expansion was only measured at the first visit for each participant and was imputed by carrying the last observation forward. Analyses were conducted using R version 4.4.3 (2025-02-28) (R: A Language and Environment for Statistical Computing, 2025). Linear mixed effects modelling was conducted using the R package lme4, version 1.1–37 (Bates et al., 2015), with *p*-values estimated using the R package lmerTest, version 3.1–3 (Kuznetsova et al., 2017).. GEE fitting was conducted using the R package geepack, version 1.3.12 (Højsgaard et al., 2006). Mixed effects proportional odds logistic regression models were fitted using the R package ordinal, version 2023.12–4.1 (Christensen, 2019).

## 3. Results

### 3.1. Descriptive statistics of research participants

Table 1 shows baseline clinical and molecular measures of the 205 PC included in this study. They averaged 65 years of age, and the majority were at FXTAS stage 3 with a moderate number of CGG repeats (mean = 87, range 77–102) and no AGG interruptions in 50% of the PC. Most PC demonstrated *FMR1* somatic expansion (more than one premutation allele; at 2.64). Due to the low frequencies of two *Apoε2* (i.e. *Apoε2/ε2*) and *Apoε4* alleles (i.e. *Apoε4/ε4*), participants with one and two *Apoε2* or *Apoε4* alleles were combined in comparing group differences in the frequencies. Only 27 PC presented with *Apoε2* alleles, and 43 PC carried *Apoε4* alleles. The frequencies of *Apoε*2 or *Apoε4* alleles were not significantly different between the three groups (*p* > 0.08). In addition, instability, CGG repeats, and *FMR1* mRNA levels did not differ significantly between carriers and non-carriers of the Apoε2 allele, nor between carriers and non-carriers of the Apoε4 allele (*p* > 0.5; data not shown). Table 2 shows the demographic and cognitive characteristics of participants (86 controls, 42 FXP−, and 163 FXP+) at last visit. FXP− were younger than the controls while FXP+ were older than both controls and FXP−. The average number of visits were 1.8/2.1/2.3 for the controls/FXP−/FXP+, respectively. The mean years elapsed between visits were 3.3/3.2/2.4 years for the controls/FXP−/FXP+, respectively. FXP+ had significantly higher incidence of hypertension at last visit compared with both NC and FXP−. FXP− had more years of education than controls while FXP+ had fewer years of education than both controls and FXP−. In addition, FXP+ showed lower scores in FSIQ, PSI, BDS-2, and dexterity compared with both controls and FXP− and lower WMI than controls. FXP+ exhibited higher frequencies of impaired WMI (≤ 80) than controls and higher frequencies of impaired FSIQ (≤ 80), PSI (≤ 80), and BDS-2 (< 14) than both controls and FXP−.

### 3.2. The effect of Apoε genotypes on MRI measurements

**Fig. 2.**
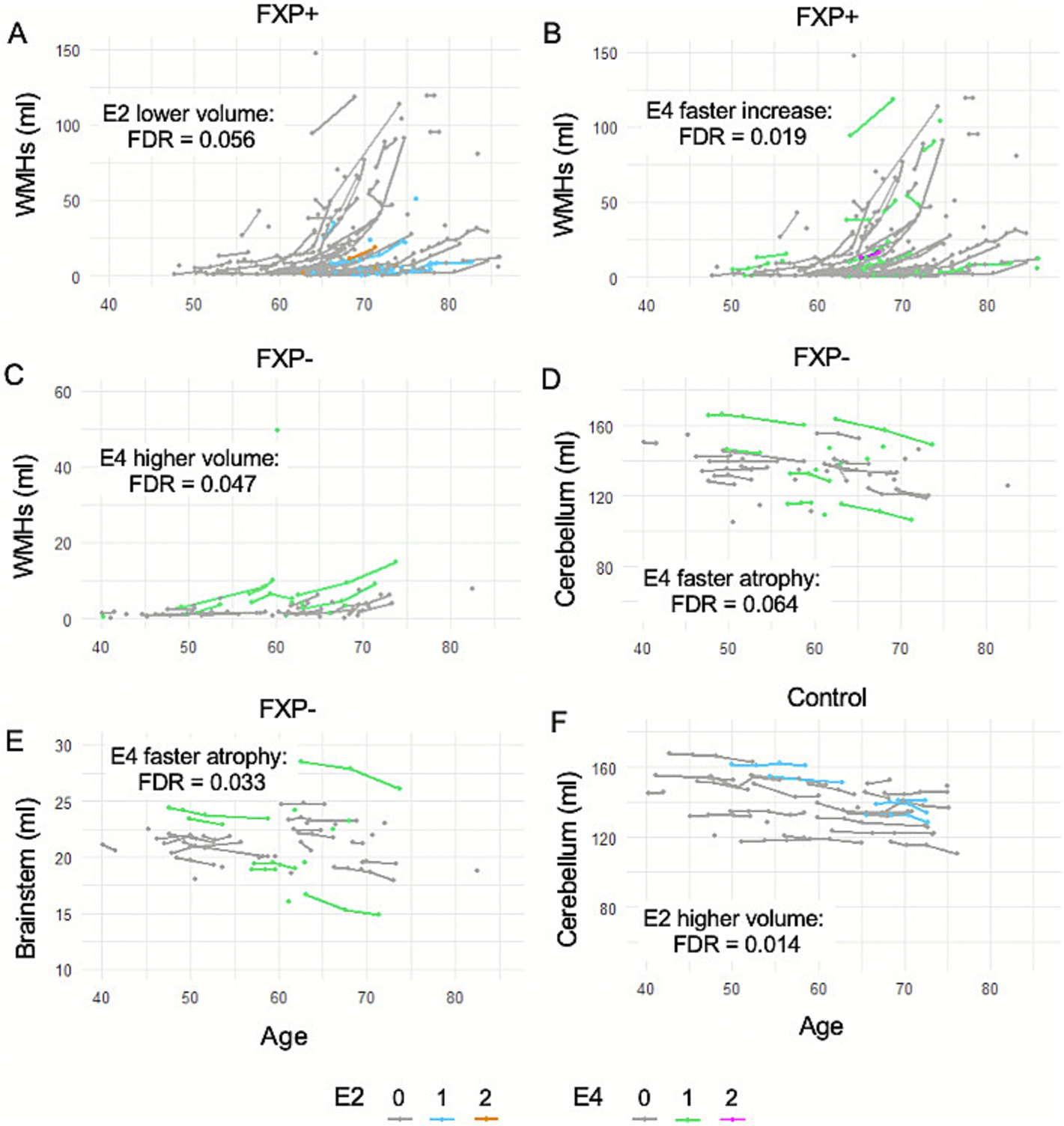
Spaghetti plots of the effect of the *Apoε2* and *Apoε4* genotypes on MRI measurements. Each dot represents a single data point of a participant. Each line links the data points of a participant indicating the trajectory overtime. (A) Premutation carriers with FXTAS (FXP+) and the *Apoε2* allele showed lower volume of white matter hyperintensities (WMHs) than FXP+ without the *Apoε2* allele. (B) FXP+ with the *Apoε4* allele showed faster increase in WMH volume than FXP+ without the *Apoε4* allele. (C) Premutation carriers without FXTAS (FXP−) and with the *Apoε4* allele showed higher WMH volume than FXP− without the *Apoε4* allele. (D) FXP− with the *Apoε4* allele showed faster cerebellar atrophy than FXP− without the *Apoε4* allele. (E) FXP− with the *Apoε4* allele showed faster brainstem atrophy than FXP− without the *Apoε4* allele. (F) Healthy controls with the *Apoε2* allele showed higher cerebellar volume than controls without the *Apoε2* allele.

We next examined the effect of *Apoε2* and *Apoε4* alleles on individual MRI measurements of neurodegeneration, including volumes of whole brain WMHs, whole brain, cerebellum, brainstem, and lateral ventricles, and the MCP sign in each of the three groups. Age, brain scaling factor, and hypertension, a known risk factor for cardiovascular diseases and white matter lesions (Moore and Ritchie, 2024) were included as covariates in the mixed-effects model. In the FXP+ group (Table 3), carriers with the *Apoε2* allele exhibited reduced WMH volume (FDR = 0.042) (Fig. 2A) and less frequency of the MCP sign (odds ratio = 0, 95% confidence interval [CI] = 0–0.001, FDR = 0.018) compared to those without the *Apoε2* allele. The percentages of FXP+ with the MCP sign were 59% (13/22) in those with the *Apoε2* allele versus 70.6% (101/143) in those without the allele. In addition, FXP+ with the *Apoε4* allele showed faster age-related increase in WMH volume (FDR = 0.020) especially at a younger age compared to those without the *Apoε4* allele (Fig. 2B). To illustrate the differences in the exponential increase in WMH volume between FXP+ with and without the *Apoε2/Apoε4* allele, we assumed no hypertension for simplicity (since the effect of hypertension on WMH volume was not significant), and an average brain scaling factor of 0.856. For FXP+ with no *Apoε2* or *Apoε4* allele*/Apoε2* allele/*Apoε4* allele, the estimated WMH volume was 3.616/1.297/5.570 ml at age 61, 4.235/1.537/6.113 ml at age 62, 4.960/1.821/6.709 ml at age 63, and increased to 20.563/8.380/15.494 ml at age 72. In the FXP− group (Table 3), carriers with the *Apoε4* allele exhibited higher WMH volume (FDR = 0.047) (Fig. 2C) and faster age-related atrophy of the cerebellum (no *Apoε4/Apoε4:* −0.53/−0.98 ml/year, FDR = 0.064) (Fig. 2D) and brainstem (no *Apoε4/Apoε4:* −0.07/−0.18 ml/year, FDR = 0.033) (Fig. 2E) compared to those without the *Apoε4* allele. In the control group, only the *Apoε2* allele showed an effect on cerebellar volume, and no other *Apoε* effects were found in this group. Controls with the *Apoε2* allele displayed higher cerebellar volume than controls without ε2 allele (*β* = 12.61 ml, SE = 3.86 ml, *p* = 0.003, FDR = 0.014) (Fig. 2F). After adding CGG repeat squared, only the reported detrimental effect of *Apoε4* allele on WMH volume remained significant in PFX+ while in PFX-, all the reported effect of *Apoε* genotypes on volumes of WMH, cerebellum, and brainstem remained significant (*p* < 0.05).

While 484/512 (95%) MRI scans were acquired between the two scanner upgrades in 2009 and 2022, respectively, 5 scans were acquired before the first upgrade, and 21 scans were obtained after the second upgrade. To eliminate the effect of scanner upgrade on the results, we repeated the analyses including only the 484 scans acquired between the two scanner upgrades. The results remained similar for the FXP+ and control groups (*p* = 0.004–0.019). For the FXP− group, the interaction between age and E4 alleles on the volumes of cerebellum and brainstem became non-significant (*p* > 0.05) while the positive effect of *Apoε4* allele on WMH volume remained significant (*p* = 0.012).

### 3.3. The effect of Apoε genotypes on cognitive function

**Fig. 3.**
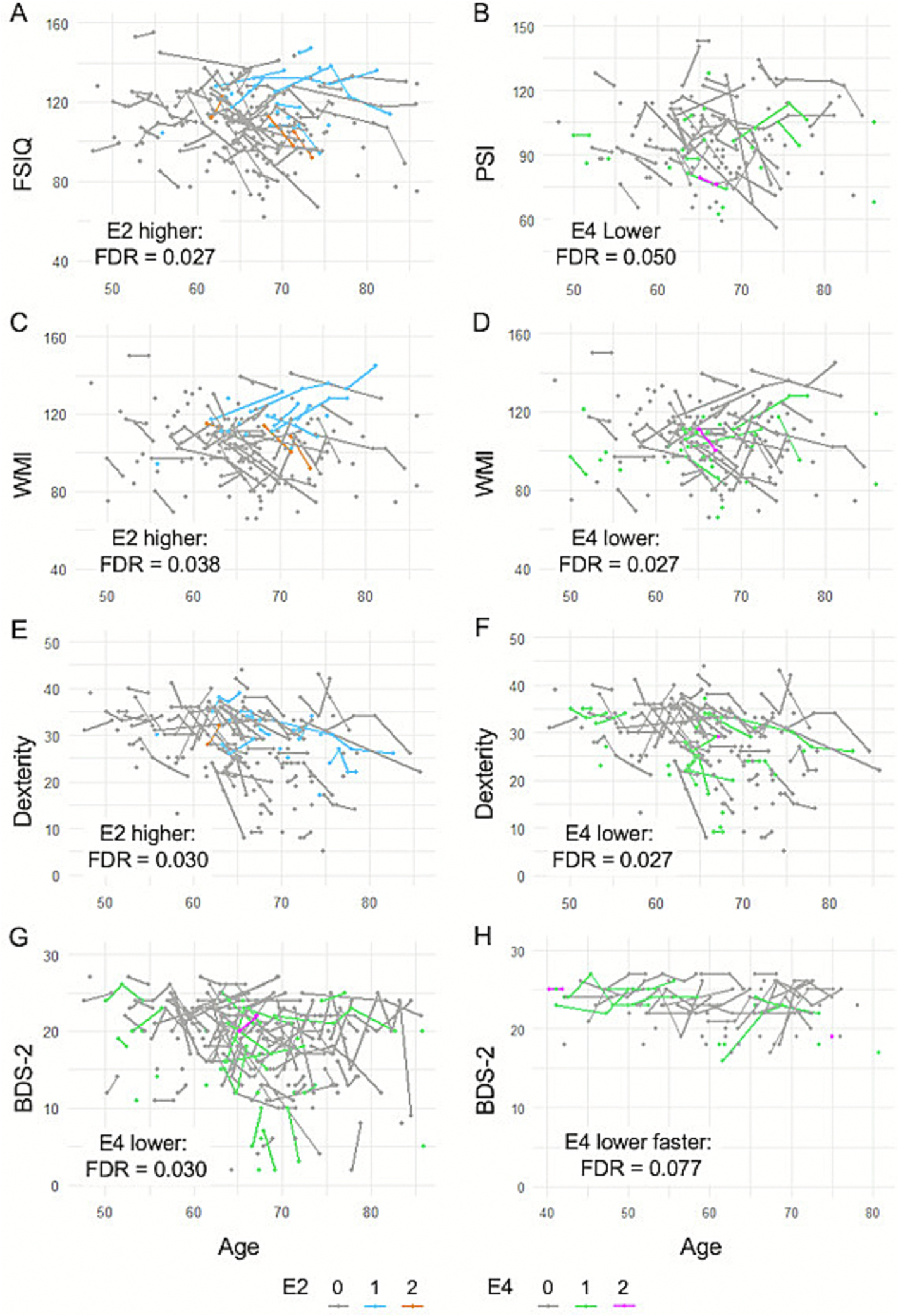
Spaghetti plots of the effect of the *Apoε2* and *Apoε4* genotypes on cognitive functions in premutation carriers with FXTAS (FXP+). Each dot represents a single data point of a participant. Each line links the data points of a participant indicating the trajectory overtime. FXP+ with the *Apoε2* allele showed higher scores of Full-Scale IQ (FSIQ) (A), Working Memory Index (WMI) (C), and dexterity (E) than FXP+ without the *Apoε2* allele. FXP+ with the *Apoε4* allele showed lower scores of Processing Speed Index (PSI) (B), WMI (D), dexterity (F) and Behavioral Dyscontrol Scale-2 (BDS-2) (G) than those without the allele. Controls with the *Apoε4* allele showed lower scores of BDS-2 than those without the *Apoε4* allele (H).

Table 4 shows the effect of the *Apoε* alleles on cognitive functions assessed using FSIQ, WMI, PSI, BDS-2, and Purdue Pegboard (for dexterity) in the FXP+ group with years of education as a covariate in the mixed effects models for FSIQ, WMI, and PSI. While FXP+ carriers with the *Apoε2* allele demonstrated better performance in FSIQ, WMI, and dexterity (FDR = 0.027–0.038) compared to FXP+ without the *Apoε2* allele, FXP+ carriers with the *Apoε4* allele exhibited worse performance in FSIQ, WMI, PSI, BDS-2, and dexterity (FDR = 0.027–0.071) compared to FXP+ without the *Apoε4* allele (Fig. 3A–G). In the controls, only the effect of the *Apoε4* allele on faster age-related decline in BDS-2 score (squared) were significant at FDR < 0.1 (*β* = −7.04, SE = 2.50, *p* = 0.006, FDR = 0.077) (Fig. 3H). To illustrate the differences in BDS-2 changes over time in the controls with and without the *Apoε4* allele, we estimated the BDS-2 scores based on the mixed-effects model. At ages 61, 62, 63, and 72, the estimated BDS-2 scores were 22.85, 22.86, 22.88 and 22.98 for a non-*Apoε4* allele carrier, respectively, and were 23.01, 22.86, 22.72, and 21.40 for a carrier of the *Apoε4*, respectively. After adding CGG repeat squared that showed significant correlation with all cognitive measures (*p* < 0.001), the effect of *Apoε* genotype remained significant for FSIQ, WMI, BDS2, and dexterity in the FXP+ group (*p* < 0.05) after adjusted for age, years of education, and IQ test version for FSIQ and WMI.

### 3.4. Somatic expansion and FXTAS stage associated with MRI measurement

Table 5 shows results of multivariable linear mixed effects models of brain volumes by somatic expansion, CGG repeats, brain scaling factor, and age at measurement. After adjusting for all other variables in the multivariable model, increased instability (*p* = 0.010) was associated with significantly higher cerebellar volume only, but not with other volume measurements or the MCP sign. Likewise, after adjusting for all other variables in the model, increased CGG repeats (*p* < 0.002) were associated with increased whole brain WMH volume, and lateral ventricle volume but decreased whole brain volume, cerebellar volume, and brainstem volume. Increased CGG repeats were also associated with increased odds of having the MCP sign (odds ratio = 1.04, 95% CI = 1.004–1.078, *p* = 0.030). Results of multivariable linear mixed effects models of brain volume by FXTAS stage (continuous), CGG repeats, and age at measurement are shown in Supplementary Table 1. After adjusting for all other variables in the multivariable model, higher FXTAS stage (*p* < 0.003), increased CGG repeats (*p* < 0.002), and older age (*p* < 0.001) were significantly associated with increased whole brain WMH volume and lateral ventricle volume, and decreased whole brain volume, cerebellar volume and brainstem volume.

Of note, we did not observe a statistically significant correlation between number of CGG repeats and presence of an *Apoε4* allele (*P* = 0.20, two-sample *t*-test) or of an *Apoε2* allele (*P* = 0.08, two-sample t-test.

## 4. Discussion

The *APOE* gene, and in particular the *APOε4* allele, is well-known to increase risk for neurodegenerative disorders. Recent reviews and case reports (Silva et al., 2013; Tassone et al., 2023) have suggested that *APOε4 may* act as a modifier gene in *FMR1* premutation carriers (increasing risk, accelerating neuropathology or cognitive decline). Although the *FMR1* and *APOε* genes are involved in distinct biological processes, and no definitive mechanistic link has been established, a mechanistic framework makes plausible that APOE-mediated pathways (Parhizkar et al., 2023) (neuroinflammation, lipid/cholesterol metabolism, synaptic/glial repair pathways) might modify the risk for FXTAS progression. Although prior studies have not specifically focused on FXTAS, the relationships between *APOε*, particularly *APOε*2, and white-matter integrity are highly relevant to brain aging and neurodegeneration. White matter, composed of myelinated axons connecting different brain regions, is crucial for efficient neural communication. Damage or loss of white matter, visible as WMHs on MRI, can slow information transfer and contribute to cognitive decline and motor problems. *APOε* isoforms influence lipid transport, myelin maintenance, neuroinflammation, and neuronal resilience, mechanisms that are central to aging-related and neurodegenerative processes. Because FXTAS is characterized by prominent white-matter pathology, insights from broader aging and neurodegeneration literature provide a biologically plausible rationale for investigating *APOε* -mediated effects as potential modifiers of FXTAS progression. Even without a direct genetic link to *FMR1* CGG repeat length, these pathways could influence individual variability in disease expression or severity.

However, the data remain preliminary, sample sizes are modest, and replication is limited (Silva et al., 2013).

In this study we aimed to investigate whether *Apoε* polymorphism is associated with brain degeneration and cognitive impairment among 205 *FMR1* male PC (age range: 40–85 years). Among FXP+ carriers, those with *Apoε2* alleles exhibited healthier brain conditions compared to those without an *Apoε2* allele. FXP+ carriers with *Apoε4* alleles demonstrated faster age-related acceleration of WMH volume especially at a younger age, compared to those not carrying an *Apoε4* allele meaning their white-matter regions showed earlier and more pronounced degeneration on MRI, which could underlie observed differences in cognitive and motor function.

FXP− with the *Apoε4* alleles demonstrated worse brain degeneration, as measured by MRI measurements, compared with those without the *Apoε4* alleles. Among all FXP+ carriers, those with *Apoε2* alleles exhibited better cognitive functioning than those without *Apoε2* alleles while those with the *Apoε4* alleles demonstrated worse cognitive functioning than those without *Apoε4* alleles. FXTAS stage, CGG repeat number, and older age were robustly associated with worse brain measures and higher stage of FXTAS increased 3-fold the odds of having the MCP sign.

Taken together, these results suggest that FXP+ who carried *Apoε2* alleles have relatively more intact brain structure and better cognitive functioning compared to FXP+ without the *Apoε2* allele, while both FXP+ and FXP− with *Apoε4* alleles exhibited worse outcomes compared to those without *Apoε4* alleles. Consistently, the association of *Apoε4* polymorphism to brain atrophy and white matter was consistent with what has been observed in both AD patients (Agosta et al., 2009; Wolk and Dickerson, 2010; Pievani et al., 2011) and healthy individuals (Lyall et al., 2020; Chaloemtoem et al., 2024; Heise et al., 2024; Veldsman et al., 2021; Lim et al., 2017). *Apoε* polymorphism is one of the most established factors for late-onset AD, where presence of an Apo*ε4* allele is a major genetic susceptibility factor while presence of an Apo*ε2* allele may reduce risk of developing AD (Farrer et al., 1997; Corder et al., 1993; Corder et al., 1994). Neuroimaging studies among AD patients demonstrated that *Apoε4* alleles may increase regional brain atrophy necessary for memory recollection (Squire et al., 2004; Raslau et al., 2015), particularly in the hippocampus and amygdala compared to AD patients without *Apoε4* alleles (Lehtovirta et al., 1995; Geroldi et al., 1999).

Although AD and FXTAS are both progressive neurodegenerative disorders with associated cognitive decline (Seritan et al., 2008), AD affects prominently short and long-term memory (McKhann et al., 2011; Jahn, 2013) in contrast to FXTAS, which is characterized primarily by motor dysfunction (Hagerman and Hagerman, 2016). Previous neuroimaging studies of individuals with FXTAS established robust associations between regional and global MRI measures of neurodegeneration including atrophy of cerebellum, brainstem, and whole brain (Wang et al., 2017), lateral ventricular enlargement (Wang et al., 2020), WMH and MCP sign (Elias-Mas et al., 2024). Interestingly, in this study, the *Apoε2* allele appeared to show a potential protective trend among FXP+ carriers, which is similar to previous studies in AD. In FXP+, presence of an *Apoε2* allele is robustly associated with reduced likelihood of the MCP sign and decreased WMH volume. In addition, it is believed to mediate lipid metabolism and catabolism (Kim et al., 2009), activate neuroprotective signaling pathways (Hoe et al., 2005; Hayashi et al., 2007), and contribute to neurotrophic effect by preserving neuronal survival and synaptic functions (Huang et al., 2019; Li et al., 2020). In addition, presence of the *Apoε2* allele is thought to play a role in longevity (Sebastiani et al., 2012; Deelen et al., 2014; Zeng et al., 2016) by up-regulating lipid metabolites measured in the peripheral blood (Karjalainen et al., 2019; Zhao et al., 2020). Interestingly, previous studies have demonstrated alterations in lipid metabolism, especially in phospholipid and sphingolipid pathways, in both plasma and fibroblasts from FXTAS participants, implicating lipid dysregulation as a potential key contributor to FXTAS pathogenesis (Song et al., 2016; Kong et al., 2019; Zafarullah et al., 2020).

This study also revealed that presence of an *Apoε4* allele was significantly associated with greater WMH volume and faster age-related atrophy of cerebellum and brainstem among FXP− carriers. These results are consistent with prior findings (Wang et al., 2017; Hashimoto et al., 2011a; Hashimoto et al., 2011b; Wang et al., 2013) that reduced cerebellum and brainstem volumes were detected before the onset of FXTAS clinical symptoms (Hagerman and Hagerman, 2016) and were independent of the CGG repeat size in FXP−. *Apoε4* involves several mechanisms that may underlie neuronal degeneration including impairment of axonal transport, neuronal plasticity, and synaptogenesis (Jagust and Landau, 2012; Huang and Mahley, 2014; Knopman et al., 2014), synaptic degeneration (Huang et al., 2019), reduced neuronal outgrowth (Nathan et al., 1994; Wang et al., 2005), and reduced spine and neurite density (Dumanis et al., 2009; Reas et al., 2024). These mechanisms may contribute to increased WMH volume and accelerated age-related atrophy of cerebellum and brainstem as demonstrated in this study. However, the underlying mechanism on how *Apoε4* affects these specific brain areas in *FMR1* carriers needs to be elucidated.

Findings from this study also revealed patterns of a positive effect of *Apoε2* and a negative effect of *Apoε4* on cognitive measures in FXP+ carriers who were mostly cognitively low average to very superior global intelligence (IQ 80–147), with only 10.6% of FXP+ showing impaired cognitive abilities (IQ ≤ 80). FXP+ with *Apoε2* alleles exhibited higher IQ, working memory, and dexterity than FXP+ without *Apoε2*. Conversely, IQ, working memory, cognitive efficiency, executive function, and dexterity are worse among FXP+ with *Apoε4* allele than those without. These results are in agreement with longitudinal investigations of healthy adults that *Apoε2* carriers exhibited decreased age-related decline in cognition (Blair et al., 2005; Shinohara et al., 2016), working memory (Weiss et al., 2021), episodic memory (Rajan et al., 2019), executive function (Reas et al., 2019), and verbal learning ability (Helkala et al., 1996). It has also been reported that in much older healthy adults (≥ 90 years) with neuroimaging biomarkers of AD, *Apoε2* alleles were associated with relatively better cognitive functioning (Berlau et al., 2007; Berlau et al., 2009). Further, prior studies of *Apoε4* alleles revealed that in a cognitively normal population, those with homozygous *ε4* genotype showed worse global cognitive functions (Christensen et al., 2008) and lower nonverbal fluency than those with a non-*Apoε4* genotype (Izaks et al., 2011).

Consistent with previous studies (Hessl et al., 2025; Wang et al., 2017; Wang et al., 2020; Wang et al., 2022), FXTAS stage, CGG repeat length, and older age were significantly associated with worse brain measures, namely, increased whole brain WMH volume and lateral ventricle volume, and decreased whole brain, cerebellar, and brainstem volume in PC. Interestingly, CGG repeat instability was significantly associated with higher cerebellar volume. CGG repeat instability has been demonstrated in the mouse models of fragile X-related disorders and other repeat expansion diseases in humans (Lokanga et al., 2013; Zhao et al., 2015). Generally, this somatic expansion required euchromatic chromatin configuration leading to an expansion and a gain of averagely 1–2 repeats per event (Møllersen et al., 2010). However, evidence demonstrated that fragile X syndrome (FXS) patient-derived induced pluripotent stem cells (iPSCs) and FXS embryonic stem cells were biased against large expansive active alleles and turned silence during neuronal differentiation (Zhou et al., 2016). This may lead to the occurrence of contractions resulting in good cerebellar conditions as seen in this study.

As previous work has established that *Apoε* genotype modulates white-matter integrity and myelin maintenance in aging and AD, our study extends this understanding to FXTAS, a disorder driven by *FMR1* CGG repeat expansions, showing that *Apoε* may act as a modifier of white-matter vulnerability, and cognitive function. These results are important because they provide the first evidence that common genetic variation can influence individual differences in FXTAS manifestation, independent of the primary mutation. This insight advances our understanding of how pathway-level mechanisms, such as *Apoε*-mediated lipid transport and myelin maintenance, shape neurodegenerative outcomes, and may inform early identification, prognosis, and potential intervention strategies in FXTAS and related disorders.

One limitation of this study that may affect the statistical analysis is the unequal representation of *Apoε* genotypes: 140 participants (68%) had Apoε3/Apoε3 alleles, whereas only 27 (13.2%) carried an Apoε2 allele and 43 (20.9%) carried an Apoε4 allele. In addition, multiple studies have reported the effect of *FMR1* premutation on the basal ganglia structure (Wang et al., 2020; Elias-Mas et al., 2024; Birch et al., 2017). We are currently obtaining the segmentation of basal ganglia accurately to expand the analysis to this important structure in FXTAS.

In conclusion, while limited by small subgroup sizes, this is the first study to suggest a possible protective effect of the *Apoε2* allele and the deleterious impact of the *Apoε4* allele on brain structure and cognitive function in PC, mirroring patterns observed in AD; in this sense, *Apoε* polymorphism may be considered a secondary genetic modifier of risk for FXTAS. Notably, *FMR1* somatic expansion was linked to increased cerebellar volume, suggesting a potentially complex interplay between genetic modifiers and disease expression.

Finally, these findings highlight the importance of assessing *Apoε* genotype and somatic expansion in PC prior to the onset of FXTAS symptoms, as early identification may support more aggressive symptom management and lifestyle modifications to improve long-term outcomes and quality of life.

## Supplementary Material

1

## Figures and Tables

**Table 1:** Baseline clinical and molecular measures of premutation carriers.

Characteristics (Hagerman et al., 2001)	*N* = 205 (Jacquemont et al., 2003)

**Age**	65 (59, 70)
**FXTAS stage**	
0	40 (20%)
1	33 (16%)
2	38 (19%)
3	56 (27%)
4	30 (15%)
5	8 (3.9%)
***FMR1* mRNA**	2.57 (2.16, 2.96)
Unknown	21
**CGG repeats**	87 (77, 102)
Unknown	12
**AGG interruptions**	
0	86 (50%)
1	58 (34%)
2	28 (16%)
Unknown	33
**Instability**	2.64 (2.03, 3.85)
Unknown	90
**ApoE**	
E2, E2	3 (1.5%) 1 (0.5%)
E2, E3	19 (9.3%)
E2, E4	5 (2.4%)
E3, E3	140 (68%)
E3, E4	37 (18%)
E4, E4	1 (0.5%)
**ApoE (E4/E2)**	
No E4 allele	162 (79%)
E4 allele present	43 (21%)
No E2 allele	178 (87%)
E2 allele present	27 (13%)

**Table 2:** Demographics and cognitive statistics of participants at last visit.

Groups	Controls	FXP-	FXP+	*P* values		
				FXP- vs. NC	FXP+ vs. NC	FXP+ vs. FXP-

Age: Mean (SD) [range] [N]	63.1 (9.9) [40–81] [86]	59.0 (11.6) [40–82] [42]	68.3 (7.4) [48–85] [163]	0.013	<0.001	<0.001
Number of visits: Mean (SD) [range]	1.8 (1.3) [1–6]	2.1 (1.4) [1–6]	2.3 (1.6) [1–10]	0.22	0.023	0.67
Years elapsed between visits: Mean (SD) [range]^a^	3.0 (1.3) [0.2–8.5]	2.9 (1.6) [0.3–7.0]	2.2 (1.5) [0.3–9.5]	0.71	0.001	0.011
Hypertension: % [N]	32.9% [70]	41.5% [41]	60.5% [162]	0.41	<0.001	0.034
Education (y): Median (IQR) [range] [N]	16.5 (3.3) [6–26] [68]	18 (4) [6–24] [40]	16 (4) [8–25] [143]	0.043	0.043	0.001
Full scale IQ: Mean (SD) [range] [N]	120.2 (14.9) [90–153] [64]	122.2 (15.2) [91–148] [39]	106.9 (18.1) [67–147] [113]	0.64	0.004	0.004
FSIQ ≤80: %	0%	0%	10.6%	1.00	0.004	0.037
WMI: Mean (SD) [range] [N]	111.0 (15.3) [82–141] [42]	114.3 (16.8) [90–147] [15]	102.9 (17.4) [66–145] [94]	0.67	0.038	0.051
WMI ≤ 80: %	0%	0%	10.6%	1.00	0.031	0.35
PSI: Mean (SD) [range] [N]	107.3 (15.5) [79–137] [43]	112.5 (15.2) [84–140] [15]	90.6 (17.8) [56–143] [75]	0.16	0.008	<0.001
PSI ≤ 80: %	2.0%	0%	28.0%	1.00	<0.001	0.018
BDS-2: Median (IQR) [range] [N]	23 (5) [17–27] [69]	23 (4.75) [13–27] [38]	19 (8) [2–27] [149]	0.68	<0.001	<0.001
BDS-2 < 14: %	0%	2.6%	24.8%	0.36	<0.001	0.001
Dexterity: median (IQR) [range] [N]	36 (9) [18–51] [62]	35.5 (7.5) [25–45] [36]	27.5 (10.5) [5–42] [100]	0.40	<0.001	<0.001

a Included all visits. Abbreviations: BDS-2, Behavioral Dyscontrol Scale-2; FSIQ, full scale IQ; FXP-, fragile X PC without FXTAS; FXP+, fragile X PC with FXTAS; IQR, interquartile range; PSI, processing speed index; WMI, working memory index.

**Table 3:** The effect of Apoε genotype on MRI measurements of neurodegeneration in PC with and without FXTAS.

Correlation	Carriers with FXTAS				Carriers without FXTAS			
	*β*	SE	*P*	FDR	*β*	SE	*P*	FDR

*WMH (log mm^3^)*	*N* = *133, # of observations* = *281*				*N* = *49, # of observations* = *88*			
Age	0.16	0.010	< 0.001	**<0.001**	0.06	0.01	< 0.001	**0.002**
E2	−1.05	0.41	0.011	**0.042**	−0.33	0.37	0.37	0.55
E4	0.42	0.34	0.22	0.47	0.80	0.31	0.013	**0.047**
E2 × age	0.014	0.026	0.58	0.72	−0.03	0.03	0.33	0.54
E4 × age	−0.065	0.022	0.005	**0.020**	0.04	0.02	0.13	0.28
*Whole brain (ml)*	*N* = *109, # of observations* = *218*				*N* = *43, # of observations* = *83*			
Age	−7.09	0.75	< 0.001	**<0.001**	−4.22	1.12	0.001	**0.004**
E2	26.0	26.5	0.33	0.52	−36.9	22.6	0.11	0.25
E4	−4.74	18.3	0.80	0.88	−4.90	19.0	0.80	0.87
E2 × age	−0.013	2.64	1.00	1.00	1.05	2.27	0.65	0.75
E4 × age	1.38	1.75	0.43	0.62	−0.22	2.03	0.92	0.92
*Cerebellum (ml)*	*N* = *109, # of observations* = *218*				*N* = *43, # of observations* = *83*			
Age	−0.78	0.11	< 0.001	**<0.001**	−0.53	0.12	< 0.001	**0.001**
E2	1.40	4.13	0.74	0.85	−2.22	4.99	0.66	0.75
E4	4.59	3.09	0.14	0.38	2.74	4.30	0.53	0.66
E2 × age	0.45	0.36	0.22	0.46	0.30	0.24	0.22	0.39
E4 × age	0.14	0.26	0.59	0.74	−0.45	0.19	0.021	**0.064**
*Brainstem (ml)*	*N* = *109, # of observations* = *218*				*N* = *43, # of observations* = *83*			
Age	−0.14	0.016	< 0.001	**<0.001**	−0.07	0.02	0.008	**0.033**
E2	−0.10	0.69	0.88	0.91	−0.68	0.90	0.45	0.60
E4	0.60	0.54	0.27	0.47	−0.08	0.78	0.92	0.92
E2 × age	0.062	0.054	0.26	0.47	0.06	0.05	0.22	0.39
E4 × age	−0.025	0.039	0.52	0.71	−0.11	0.04	0.007	**0.033**
*LV (log ml)*	*N* = *109, # of observations* = *218*				*N* = *43, # of observations* = *83*			
Age	0.046	0.003	< 0.001	**<0.001**	0.04	0.00	< 0.001	**< 0.001**
E2	−0.15	0.14	0.26	0.47	0.15	0.18	0.40	0.56
E4	0.068	0.111	0.54	0.71	0.15	0.15	0.34	0.54
E2 × age	−0.020	0.010	0.042	0.13	−0.02	0.01	0.05	0.14
E4 × age	0.006	0.007	0.39	0.58	0.01	0.01	0.11	0.25
*The MCP sign*	*N* = *164, # of observations* = *345*				–			
Age	0.30	0.23	0.19	0.47	–	–	–	–
E2	−21.6	7.42	0.004	**0.018**	–	–	–	–
E4	−0.20	2.14	0.93	0.96	–	–	–	–
E2 × age	1.12	0.60	0.06	0.18	–	–	–	–
E4 × age	−0.32	0.30	0.29	0.48	–	–	–	–

Bold, FDR ≤ 0.10. Abbreviations: FDR, false discovery rate; LV, lateral ventricles; MCP middle cerebellar peduncle; WMH, white matter hyperintensity.

**Table 4:** The effect of *Apoε* genotype on cognitive functions in FXP+.

Correlation	*β*	SE	*P*	FDR

*FSIQ*	*N* = *135, # of observations* = *233*			
E2	9.70	2.74	0.007	**0.027**
E4	−5.19	−1.92	0.06	**0.071**
*WMI*	*N* = *132, # of observations* = *197*			
E2	9.12	2.30	0.023	**0.038**
E4	−8.29	−2.69	0.008	**0.027**
*PSI*	*N* = *105, # of observations* = *161*			
E2	5.88	1.44	0.15	0.17
E4	−7.42	−2.14	0.035	**0.050**
*BDS-2 squared*	*N* = *158, # of observations* = *318*			
E2	38.43	1.00	0.32	0.32
E4	−80.22	−2.46	0.015	**0.030**
*Dexterity*	*N* = *119, # of observations* = *228*			
E2	5.05	2.53	0.013	**0.030**
E4	−4.43	−2.77	0.007	**0.027**

Bold, FDR ≤ 0.10. Abbreviations: BDS-2, Behavioral Dyscontrol Scale-2; FSIQ, full scale IQ; PSI, processing speed index; WMI, working memory index.

**Table 5:** Multivariable linear mixed-effects models of brain volume by CGG repeat instability and CGG repeat length in PC adjusted for age and cranial size.

MRI outcome measures	*N (# of observations)*	Log_2_(CGG repeat instability)			CGG repeats		
		Fold change*	95% CI	*P*-value	Fold change*	95% CI	*P*-value

WMH (log mm^3^)	108 (242)	1.024	0.835, 1.256	0.80	1.039	1.023, 1.054	**<0.001**
Whole brain (log l)	83 (195)	1.019	0.997, 1.041	0.09	0.998	0.997, 0.999	**<0.001**
Cerebellum (log ml)	83 (195)	1.061	1.015, 1.109	**0.010**	0.994	0.992, 0.996	**<0.001**
Brainstem (log ml)	83 (195)	1.033	0.978, 1.091	0.20	0.995	0.993, 0.998	**0.002**
Lateral ventricles (log ml)	83 (195)	0.874	0.716, 1.067	0.20	1.019	1.009, 1.029	**<0.001**

Bold, *p* < 0.05.

* Fold change for a continuous variable [i.e. log_2_(CGG repeat instability) or CGG repeat length] is the multiplicative change in the indicated brain measure for a unit increase in the continuous variable.

## Data Availability

Data will be made available upon request.

## Appendix A. Supplementary data

Supplementary data to this article can be found online at https://doi.org/10.1016/j.nbd.2026.107292.

## Glossary

FXTAS: Fragile X-associated tremor/ataxia syndrome
FMR1: fragile X messenger ribonucleoprotein 1 gene
WMH: white matter hyperintensities
Apoε: Apolipoprotein E
MCP: middle cerebellar peduncle
LV: lateral ventricles
PC: premutation carriers
PVS: perivascular spaces
AD: Alzheimer's disease
FXP+: premutation carriers with FXTAS diagnosis
FXP: premutation carriers with no FXTAS diagnosis
FSIQ: Full scale IQ
WMI: Working Memory Index
PSI: Processing Speed Index
BDS-2: Behavioral Dyscontrol Scale-Second Edition
FDR: Benjamini-Hochberg's false discovery rate
