# Supplementary material for: The apolipoprotein gene: a modulating role on brain volume and cognitive function in carriers of the fragile X premutation
Source: Neurobiol Dis. Author manuscript; Available in PMC 2026 Jul 2. (PMC13325199; doi:10.1016/j.nbd.2026.107292)
Supplement: 1 [file NIHMS2167346-supplement-1.docx]

**Supplementary table 1** Multivariable Linear Mixed Effects Models of Brain Volume by FXTAS stage, CGG repeats, and age, premutation subjects.

| **MRI outcome measures** | ***N* (number of observations)** | **FXTAS stage** | | | **CGG repeats** | | | **Age at brain scan** | | |
| --- | --- | --- | --- | --- | --- | --- | --- | --- | --- | --- |
|  |  | **Fold change*** | **95% CI** | ***P*-value** | **Fold change*** | **95% CI** | ***P*-value** | **Fold change*** | **95% CI** | ***P*-value** |
| WMH (log mm^3^) | 157 (360) | 1.291 | 1.203, 1.385 | **<0.001** | 1.026 | 1.018, 1.035 | **<0.001** | 1.105 | 1.091, 1.120 | **<0.001** |
| Whole brain (log l) | 127 (292) | 0.993 | 0.989, 0.997 | **0.001** | 0.999 | 0.999, 1.000 | **<0.001** | 0.997 | 0.996, 0.998 | **<0.001** |
| Cerebellum (log ml) | 127 (292) | 0.991 | 0.985, 0.997 | **0.003** | 0.999 | 0.998, 1.000 | **0.002** | 0.995 | 0.994, 0.996 | **<0.001** |
| Brainstem (log ml) | 127 (292) | 0.987 | 0.981, 0.993 | **<0.001** | 0.999 | 0.998, 0.999 | **0.001** | 0.995 | 0.994, 0.996 | **<0.001** |
| Lateral ventricles (log ml) | 127 (292) | 1.046 | 1.027, 1.065 | **<0.001** | 1.005 | 1.002, 1.008 | **<0.001** | 1.038 | 1.034, 1.042 | **<0.001** |

Bold, p < 0.05. *Fold change for a continuous variable (i.e. FXTAS stage, CGG repeat instability or age) is the multiplicative

change in the indicated brain measure for a unit increase in the continuous variable.
